# Adolescents' experiences of the public health nurse's accessibility, guidance, and counseling about sexuality: a qualitative study

**DOI:** 10.3389/fpubh.2026.1825174

**Published:** 2026-07-23

**Authors:** Silje Engen Johannessen, Gregory Luc Romain Mauquet, Ragnhild Sollesnes, Helene Åvik Persson

**Affiliations:** 1 Department of Health and Caring Science, Faculty of Health and Social Sciences, Western Norway University of Applied Sciences, Bergen, Norway; 2 Preventive Health Services for Children and Young People, Kongsberg Municipality, Norway; 3 Department of Health Sciences, Faculty of Medicine, Lund University, Lund, Sweden

**Keywords:** accessibility, adolescents, counseling, guidance, public health nurse, sexuality

## Abstract

**Introduction:**

Public health nurses play a central role in health promotion and preventive care and are involved in various aspects of adolescent health, including sexuality. Adolescents' sexuality has changed significantly over the past few decades and is characterized by an increase in abortion and sexually transmitted infections. These developments have potentially wide-ranging consequences for adolescents' health at both individual and societal levels. Therefore, this study aimed to explore adolescents' experiences of the public health nurse's accessibility, guidance, and counseling concerning sexuality.

**Methods:**

A qualitative, explorative design with an inductive approach was applied. Data collection was carried out in 2024–25 using individual interviews based on a semi-structured interview guide. A total of eleven girls aged 16–20 who had been in contact with public health nurses in their school health service or at a youth health center for guidance and counseling related to sexuality were included in the study. The data material was analyzed in accordance with Graneheim and Lundman's qualitative content analysis.

**Results:**

The overarching theme, key elements influencing adolescents' contact with public health nurses, summarizes three main categories: a focus-oriented service offered for adolescents' use, the dynamics of the interaction, and public health nurses' role, followed by six subcategories.

**Conclusion:**

The engagement of adolescents with public health nurses in relation to sexual health issues is influenced by an interaction between accessibility, relational quality and the role of public health nurses in sexual health education. Collectively, these factors appeared to support health literacy and the process of help-seeking. An increase of the public health nurse's visibility and involvement in school settings has the potential to further facilitate adolescents access to support based on trust and sexuality-related guidance. Public health nurses also play a key role in sexual education, underscoring the importance of close collaboration between youth health services and upper secondary schools to effectively support adolescents' needs and wellbeing.

## 1 Introduction

Universal access to sexual and reproductive healthcare services is explicitly emphasized in two of the United Nations' (UN's) Sustainable Development Goals within Agenda 2030 (1). Despite this global commitment, adolescents continue to face substantial challenges related to sexual and reproductive health and rights, and progress in this area remains insufficient (2, 3). Adolescence represent a transitional phase between childhood and adulthood and is characterized by rapid physical, cognitive, emotional, and sexual development (4–6). Sexuality is an integral part of human life across the lifespan, beginning at birth, and is a central aspect of public health (7).

Over the past few decades, societal changes, including shifts in youth culture and broader social conditions, have further influenced adolescents' health-related behaviors, lifestyles, and overall health status (8). Sexual and reproductive health has become a key public health issue in this population. Research highlights concern about early sexual debut, inadequate use of contraception, and insufficient information which may lead to unwanted pregnancies, sexually transmitted infections, and mental health challenges (1, 9–11). A Norwegian study among high school students who had debuted sexually, found that about half reported their first sexual intercourse before age of 16 (11). Scandinavian research indicates a slight decline in the age of sexual debut, from 17 to 16 years (9). International findings further show that approximately 20% of young people report their first sexual intercourse as unwanted, with negative experiences more frequently reported among girls (10). In addition, condom use among European youth has declined in recent years (12), that there is no clearly established culture of condom use (13). In a Norwegian high school study, 32% reported not using contraception at first intercourse, with higher use among girls than boys (11).

As a result of these developmental changes, adolescents have distinct health needs that differ from those of both younger children and adults, and these should be addressed with consideration of their development stage (5, 14). Therefore, for adolescents to grow and develop in good health, it is essential to have access to information, age-appropriate sexuality education, opportunities to develop life skills, acceptable health services, and safe environments (15).

Schools are increasingly recognized as key settings for promoting and protecting adolescent health and wellbeing. They provide opportunities to acquire knowledge, develop socio-emotional skills such as self-regulation and resilience, and strengthen critical thinking skills, all of which are fundamental to ensuring a healthy future (14). Within this context, public health nurses (PHN) play a central role in health promotion and preventive work, including efforts related to sexuality. Their responsibilities include strengthening adolescents' autonomy and coping skills, providing individual guidance and counseling, and contributing to health education in groups and classroom settings (16, 17).

In Norway, youth health services (YHS), including school health services (SHS) and youth health centers (YHC), which are intended to supplement SHS, are legally mandated (16). Every municipality is required to provide these services which are responsible for promoting the health and wellbeing of school-aged children and adolescents (16, 18).

The professionals providing these services are PHNs, who are registered nurses with a further 1, 5 year postgraduate study or two-year master program in public health nursing (19, 20). The PHN's role corresponds to that of the school nurse, but because PHNs also work at YHCs, the term “public health nurse” is used in this study (21).

Previous research has examined the accessibility of school nurses in several different areas; for example, in situations where school nurses identify students who are being exposed to violence (22). Accessibility has also been identified as an important aspect of school nurses' work in health promotion in areas such as stress management (23). Identified facilitators to adolescents' access to and positive experiences of sexual health counseling are actions such as clear confidentiality policies, optional meeting environment, either in person or digitally, youth-friendly opening hours, active information provision, and staff training (24, 25). However, several recurring barriers have also been observed including concerns about confidentiality, stigmatization, low awareness of available services, cultural norms, and lack of time and education among staff (26). Within a Nordic context, empirical studies add further nuance to these findings by illustrating how structural conditions and relational processes may jointly influence how adolescents' experiences of, engagement with, and access to school health services (27, 28). A Finnish study investigating access to school health nurses within the universal school health service in Finland found that most adolescents agreed that access was good (28). However, a proportion found access difficult, and there was a call for further research into barriers to accessing school health services. At the same time, Swedish research (27) emphasized the importance of the relation dimension of school nurses, showing that establishing a caring relationship with students is a prerequisite for caring and health promotion. In relation to sexual health, recent findings (29) indicate that structured approaches support school health professionals in normalizing conversations about sensitive topics and facilitating the identification of adolescents in need of support. These approaches may also be integrated as part of routine preventive work within SHS. However, organizational and structural barriers may still limit implementation (29). In a Norwegian context, (PHN)s emphasize that their role depends on both physical and digital availability, while also highlighting the importance of presence within the school setting and interprofessional collaboration (30). Taken together, a recent scoping review, published in 2026 (31), which examined the school nurse's contribution to foster and promote a positive sexuality for adolescents, highlights together with above previous research (27–30) a clear need for further research to strengthen the promotion of sexual health among young people. Among other factors, the review emphasized the availability and visibility of school nurses as key elements influencing the potential areas for improvement in school-based sexual health guidance and counseling (31).

These inconsistencies in literature point to a broader need for research that moves beyond identifying structural barriers and facilitators to examine how adolescents experience and perceive PHNs accessibility, guidance and counseling concerning sexuality. Such knowledge is valuable because an understanding of adolescents contemporary life contexts is essential for promoting sexual and reproductive health and rights and to address the diversity among adolescents (32). In light of this, further research is needed to deepen the understanding of adolescents' experiences. Therefore, this study aimed to explore adolescents' experiences of PHNs' accessibility, guidance, and counseling concerning sexuality.

## 2 Methods

### 2.1 Study design

The study applied an exploratory, qualitative design with an inductive approach, because this type of design is well suited to describing, analyzing, and interpreting human experiences and phenomena (33). Data was collected through individual, semi-structured interviews and analyzed using qualitative content analysis inspired by Graneheim and Lundman (34, 35). The Consolidated Criteria for Reporting Qualitative Research (COREQ) (36) were followed when presenting this study.

#### 2.1.1 Setting

SHS and YHCs are statutory services for adolescents (18), regulated by national professional guidelines (17), and anchored in the regulations on health centers and school health services (16). Within these services, the PHN plays a central role in ensuring adolescents' right to universal, free, and easily accessible healthcare until the age of 20, with services adapted to their individual needs (16, 17). According to regulations §5 and §6 (16), the PHN must contribute to strengthening adolescents' autonomy and ability to cope with everyday life, including matters related to sexual health. They must also offer guidance and counseling on sexuality adapted to each individual's needs.

### 2.2 Sampling

Purposive sample was used, which involved selecting the most beneficial cases for the study (37). Adolescents aged 16–20 who had been in contact with a PHN in Norway for guidance and counseling about sexuality were recruited. Adolescents who could contribute varied and in-depth insights were selected, and experience with the phenomenon to be investigated was considered a key prerequisite (37).

Adolescents who had been in contact with a PHN at school or at a YHC in Norway for guidance and counseling on sexuality were recruited via PHNs working in both rural and urban areas. Recruitment was carried out by PHNs, who disseminated information about the study to potential participants, providing them with information about the study and contact information for the authors. They could then establish contact if they wished to participate in the study. Exclusion criteria included adolescents who did not speak Norwegian, as well as those who had not been in contact with a PHN for guidance or counseling in a Norwegian context. In Norway, PHNs working for school health services and YHCs follow national guidelines that are specifically designed for these services (17).

To facilitate broad geographical representation, adolescents from both rural and urban areas were included in the sample, which also showed good variation in terms of age and geographic affiliation. A total of eleven female adolescents (16–20 years) were included in the study (Table 1).

**Table 1:** Overview of sociodemographic characteristics.

Sociodemographic characteristics, *n*=11	
Age (years)	
16	1
17	5
18	3
19	1
20	1
Gender	
Female	11
Location	
Urban	5
Rural	6

### 2.3 Data collection

Data collection was conducted between November 2024 and January 2025 and consisted of a total of eleven semi-structured interviews. The interview guide was tested through mutual rehearsals and pilot interviews, to ensure thorough preparation and to strengthen interview technique (38). The interviews were conducted by first and second author, face to face, seven with individuals and two involving two adolescents each, in line with their own wishes. The interviews were audio recorded and the duration of the interviews ranged from 12 to 30 min, with an average length of 18 min. The interview guide was designed according to the principles of the semi-structured qualitative research interview (37) and was developed through a participatory process involving user involvement and two pilot interviews with adolescents. There was no previous relationship between the interviewers and the participants.

The use of a semi-structured interview guide with open-ended questions allowed the adolescents to express their thoughts and experiences freely, without being limited by predefined answer options (37, 38). The interview guide was designed to cover various aspects of adolescents' experiences of guidance and counseling about sexuality by a PHN (Supplementary Interview gudie). The guide included questions about the participants' backgrounds as well as ten open-ended main questions. Examples of questions are: “Can you talk about receiving counseling and guidance about sexuality from a PHN?”; “What is important to you when you receive advice and guidance about sexuality from a PHN?”; and “How did you experience the PHNs guidance and counseling?” These were supplemented with suggestions for follow-up questions intended to elicit more detailed information. Examples of follow-up questions are: “Can you tell me more?”; “Can you elaborate?”; and “In what way?”

### 2.4 Data analysis

The interviews were transcribed verbatim and consecutively. Each transcribed text was reviewed repeatedly to ensure that it was correct in all content. They were then read thoroughly with the aim of achieving a comprehensive understanding of the material. The data was analyzed using Graneheim and Lundman's qualitative content analysis (35), which is often used in health and nursing research. This approach involved both description and interpretation of the experiences shared during the interviews, i.e. both manifest and latent content (34).

Relevant sections of text that responded to the aim were identified and divided into meaning units. In a second stage, these were condensed. Thereafter, the condensed meaning units were coded. The different codes were compared based on their differences and similarities and sorted into six subcategories and three main categories. Later, a theme was abstracted (34, 35). The analysis was carried out manually and then converted into digital form in the various categories to provide a clear overview. The analysis was carried out by the first (S.E.J.) and second (G.L.R.M.) authors, together with an ongoing discussion with the last author (H.Å-P.) throughout the whole process to reach consensus. As a final check to confirm the analysis, one of the authors (R.S.) took part in the analysis process by reading the results and interpretations of the findings. Examples of the analytical procedure are given in Table 2. Quotations have been provided in the results section, as representations of the findings. These have been translated from Norwegian by the authors.

**Table 2:** Example of the analysis process.

Meaning unit	Condensed meaning unit	Code	Subcategory	Category	Theme
That it was free […]. It means a lot for freedom and security, not least, both in terms of contraception in one form or another and testing if you're nervous about it or feel symptoms or whatever it might be.	Free and easily accessible contraception and testing provide freedom and security, especially when you are nervous about symptoms.	Free services enhance autonomy and a sense of safety.	Public health nurse availability	A focus-oriented service for adolescent use	Key elements influencing adolescents' contact with public health nurses
Even though you don't just have to talk about sex and identity or sexuality, you can also go to them to talk about problems at home or drama at school or various mental health problems, which is also very reassuring. So, then I formed a very open and good view of public health nurses	You can talk to a health nurse about more than just sex and identity. They are also available to talk about problems at home, drama at school and mental health issues, which is very reassuring. This gives an open and positive view of the public health nurse.	Broad topics and accessible support enhance confidence and role perception.	Holistic approach in conversations	The dynamics of interaction	
The guidance is good, but perhaps you could have had more about it in class, instead of having to physically go to the public health nurse. You know very little about it, when you're aware of it you would like to know more about it. I mean more about it, more talk about it, more knowledge about it.	The guidance is good, but there could be more teaching about it in class. When you are ready, you would like to know more and have more knowledge.	Classroom teaching promotes sexual health knowledge and health literacy.	Education in the classroom about sexuality	Public health nurses' role	

### 2.5 Ethical considerations

This study was carried out in accordance with the World Medical Association's Declaration of Helsinki (39), the requirements of the Health Research Act (18), and the Personal Data Act (40). The study adhered to the ethical guidelines of Western Norway University of Applied Sciences, and all data was handled accordingly (41). Initially, ethical approval was sought from the Central Norway Regional Ethics Committee for Medical Research (REK) (reference number 780016). REK considered that the study did not fall under the Health Research Act and could be conducted without the need for approval. The study was reported to the Norwegian center of the knowledge sector's service provider (SIKT) (reference number 358567). SIKT found the project to be in line with privacy laws and data treatment. These research ethics assessments were essential to ensure the rights and welfare of the adolescents involved. Informed consent to participate was obtained from all participants in the study. Before the study began, the participants received both oral and written information about its purpose, the confidential treatment of the data, and the voluntary nature of participation. This included the ability to withdraw from participation at any time without further explanation (18).

## 3 Results

### 3.1 Theme and summary of the findings

The analysis yielded one theme, three main categories, and six sub-categories (Table 3). The overarching theme encompasses several key elements influencing adolescents' contact with PHNs. It also reflects the underlying meaning that adolescents' experiences of accessibility, guidance, and counseling concerning sexuality are shaped by the interaction between structural, relational, and professional dimensions. A focus-oriented service for adolescent use, the dynamics of interaction, and the role of PHNs jointly illustrate how accessibility, trust, and professional presence influence adolescents' opportunities and willingness to seek guidance and counseling related to sexuality and sexual health.

**Table 3:** Overview of theme, main categories and sub-categories.

Theme		
Key elements influencing adolescents' contact with public health nurses		
**Main category**	**Main category**	**Main category**
A focus-oriented service for adolescent use	The dynamics of interaction	Public health nurses' role
**Subcategory**	**Subcategory**	**Subcategory**
Public health nurses' availability	Aspects of interpersonal communication	Education in the classroom about sexuality
Gender of the public health nurse	Holistic approach in conversations	Professional competence

### 3.2 A focus-oriented service for adolescent use

The adolescents highlighted that accessibility, and the convenient location of YHS played a central role in their willingness to seek guidance and support, particularly when support was needed in matters related to sexuality. The gender of the PHN emerged as a key element shaping adolescents' comfort and openness in discussing sexuality, with a preference for female nurses facilitating trust and a sense of safety. Together, these factors reveal that both structural accessibility and relational considerations are crucial in enabling adolescents to engage with PHNs and to support their help-seeking.

#### 3.2.1 Public health nurses' availability

Experiences with availability varied, but a majority of the adolescents described YHS as easy to access, characterized by a quick response and a welcoming reception from the PHN. The location also influenced how quickly they sought help and how comfortable they felt about approaching YHS. At the same time, it was highlighted that the services should not be too visible. This contributed to a sense of safety, especially when they were concerned about symptoms. A discreet location was considered essential for preserving anonymity and protecting privacy, reducing the risk of others discovering that they were seeking help. The fact that the services were free of charge was described as a fundamental condition for feeling both freedom and security. Without financial barriers, adolescents felt more able to seek timely guidance and counseling related to sexuality.

*That it was free […]. It means a lot for freedom and security, not least, both in terms of contraception in one form or another and testing if you*'*re nervous about it or feel symptoms or whatever it might be. (Adolescent 2, 20 years)*

In contrast, some adolescents had experienced YHS as difficult to reach. Limited opening hours, which often coincided with school hours, created practical obstacles to seeking support. This inaccessibility was described not only in terms of physical presence but also as a lack of alternative communication channels. This contributed to an impression of YHS as a type of “special service” that it could be challenging to access in practice, resulting in frustration and a sense of lacking support when support was needed.

*It was very difficult to get a way to talk to her then, you were very lucky when there was someone there, otherwise it was always busy. (Adolescent 11, 17 years)*

The visible presence of PHNs within the school setting emerged as an important prerequisite for knowing where to turn for support. When PHNs introduced themselves in the classroom, this increased adolescents' familiarity with the YHS and strengthened perceptions of accessibility and safety.

*The public health nurse could have been in the classroom a little more and talked about it a bit. It would have been a good idea if more people had really realized that they could come to the public health nurse and talk about it. (Adolescent 4, 17 years)*

#### 3.2.2 Gender of the public health nurse

The gender of the PHN was also significant in shaping the adolescents' comfort when receiving guidance and counseling about sexuality. While they generally expressed openness to speaking with both male and female PHNs, many described a preference for a female PHN. This preference was linked to the belief that women more easily understood their experiences, which contributed to conversations feeling more relatable, comfortable, and safe. Although male PHNs were not dismissed as untrustworthy, discussions about sexuality were perceived as easier and less embarrassing with someone of the same gender.

*I also often find it more comfortable with women than men, at least when it comes to sexuality. I really just feel that women understand it better because they*'*ve been through a lot of the same things. It*'*s not that I feel like you can't trust men or anything like that, but I just find it more comfortable to talk to women […]. I also find it a bit less embarrassing to talk to women. (Adolescent 7, 18 years)*

### 3.3 The dynamics of interaction

The adolescents pointed out that various aspects of communication with PHNs shaped their interactions and experiences. Empathy, expressed through understanding and support, together with key communication skills such as openness, attentive listening, and involvement, conveyed a holistic approach. The fact that the PHN showed respect for adolescents' self-determination contributed to a sense of safety and wellbeing.

#### 3.3.1 Aspects of interpersonal communication

The adolescents described PHNs with good qualities as being understanding, reassuring, and helpful, with encounters characterized by patience and support rather than judgment. Feeling accepted and treated without moral scrutiny appeared to be central in enabling adolescents to speak openly and maintain a sense of autonomy in conversations about sexuality.

*I guess it's just that I don't really feel properly acknowledged, maybe. Like, if I'd gone to my family, it would probably be like, “Oh, you did that? Oh no, now you're not accepted anymore,” maybe. But if you talk to the public health nurse, it's more like, “Yeah, that's totally fine,” and they kind of take care of you. (Adolescent 6, 18 years)*

Adolescents' confidence in seeking guidance and counseling was closely linked to the relational tone established during the interaction. Uncertain or negative responses from a PHN could undermine an adolescent's motivation to engage, illustrating how the perceived quality of communication shaped their willingness to seek support. Experiences of inadequate or hesitant responses left a lasting impression, particularly in situations where adolescents were already feeling unsure or vulnerable.

“*To be honest, I don't really know what to say, I'm not sure,” was the response I got. It was quite unpleasant and demotivating. […] Strong impression on me. […] Which was very sad in a way because I was very unsure at the time. That little comment in a way has been a bit challenging. (Adolescent 3, 18 years)*

Conversations with the PHN were often experienced as free, open, and informal, helping to reduce feelings of discomfort. Being seen, heard, and listened to created space for self-expression and strengthened the sense of being taken seriously. Although sexuality was initially described as an embarrassing topic, discomfort gradually shifted toward a more positive experience when the PHN communicated openly and maintained a calm atmosphere.

*What's important is that there's someone who listens and lets me talk in a way and who can give me feedback and answers afterwards. And then that you're open and just manage to have a normal conversation, that it's not like an interview with lots of questions, but that you can talk a little bit about it maybe. Also, that there's a certain openness, so you don't have to be embarrassed about what's going on and things like that. (Adolescent 7, 18 years)*

Frustrations arose when conversations were brief or marked by short, superficial questioning. Such interactions were interpreted as a lack of interest or time on the part of the PHN, creating a sense that the adolescents' needs were being downplayed or overlooked. This contributed to feelings that important topics were dismissed or closed down prematurely, weakening relational trust and reducing opportunities for the adolescents to articulate their concerns.

*I think some public health nurses try to finish pretty quickly, ask pretty quick questions and don't take enough time to kind of hold the conversations, to kind of ask deeper questions. So, I think sometimes it seems like they're really short on time and have to finish quickly. (Adolescent 4, 17 years)*

#### 3.3.2 Holistic approach in conversations

The value of a holistic approach was also emphasized, with the adolescents explaining that the opportunity to discuss a broad range of psychosocial issues, not only sexuality, strengthened their overall trust in PHNs. Being taken seriously across different topics reinforced a sense of safety and wellbeing. They said that it was reassuring to approach a PHN with any concern and encounter openness and support.

*Even though you don't just have to talk about sex and identity or sexuality, you can also go to them to talk about problems at home or drama at school or various mental health problems, which is also very reassuring. So, then I formed a very open and good view of public health nurses. (Adolescent 3, 18 years)*.

Conversely, when sexuality was treated as a separate or isolated topic, this created discomfort. A clear distinction between sexuality and other health issues disrupted the natural flow of conversation and made the topic appear more sensitive than necessary.

*That you distance sexuality from other bodily things you want to talk about and that distance, I think, is unnecessary and can be uncomfortable to a certain extent. (Adolescent 2, 20 years)*

### 3.4 Public health nurses' role

These adolescents' experiences suggest that PHNs play a central role in supporting their wellbeing and learning about sexuality. The PHNs' professional competence and knowledge were perceived as key resources that strengthened trust and confidence. Meanwhile, the need described by the adolescents for more information through classroom education highlights the ongoing importance of accessible and knowledgeable guidance. Together, the adolescents relied on the expertise and supportive presence of PHNs to feel informed, secured, and empowered.

#### 3.4.1 Education in the classroom about sexuality

The need for increased classroom involvement by PHNs was emphasized, particularly in relation to sex education and efforts to strengthen adolescents' health literacy. The adolescents noted that formal sex education generally ends in middle school, despite this being a period marked by significant changes. Age-appropriate education was described as essential, because growing older brought new questions and concerns that required more knowledge about the body and its development. Ongoing classroom visits were viewed as valuable, and the presence of a PHN, with their professional expertise, was valued as especially important when discussing these topics.

*The guidance is good, but perhaps you could have had more about it in class, instead of having to physically go to the health nurse. You know very little about it, when you're aware of it you would like to know more about it. I mean more about it, more talk about it, more knowledge about it. (Adolescent 11, 17 years)*

The limited presence of the PHN in the classroom made sexuality a less natural topic to discuss. A more consistent classroom presence, whether through regular visits or short sessions, was described as a way to normalize the topic and make it easier to talk about.

*There are far too few of them in the classroom […]. And there's far too little talk about it, I think maybe they should have come in […]. It should sort of normalize it, that we talk more about it […]. I think it would be more normalized if the public health nurse was more in the classroom and maybe had an hour or two a week or a month about it or just showed up a little more. (Adolescent 8, 19 years)*

#### 3.4.2 Professional competence

Professional guidance and counseling were perceived as reliable sources of thorough and trustworthy information, and the adolescents had great confidence in the PHNs' expertise. The information provided was described as safe and trustworthy, especially when compared to uncertain sources such as the internet. This strengthened feelings of safety, and access to qualified counseling and guidance, particularly regarding contraception, was considered highly valuable. Balanced information on the advantages and disadvantages of contraception was viewed as essential for understanding potential consequences and making informed decisions. Such guidance and counseling underscored the importance of having both qualified nurses and accessible information.

*I actually think it's very important and reassuring because then I know that if there's something I'm unsure about, I can talk to some public health nurses, instead of having to google or call the doctor and then get on to someone else. So, I actually think it's very reassuring and that it's so accessible that I can only come if I know there's something I'm wondering about. It's nice to also be able to talk to some professionals that you can trust. (Adolescent 7, 18 years)*

At the same time, unmet expectations regarding PHN expertise could weaken trust. When responses lacked clarity or completeness, participants described the experience as unsettling and embarrassing.

*I didn't get a complete answer to that, so I felt a little empty and a little embarrassed because I've always had the perspective that public health nurses can do almost anything. (Adolescent 3, 18 years)*

## 4 Discussion

The findings of this study highlight central elements concerning how adolescents experience PHNs' accessibility, guidance, and counseling in relation to sexuality. Overall, the results highlight the importance of relational, structural and professional conditions that enable adolescents to perceive these services as supportive and relevant. When accessibility, guidance, and counseling are experienced as consistent and responsive to adolescents' needs, they appear to contribute to a strengthened sense of confidence and being able to access assistance in navigating issues related to sexuality.

A central finding of this study was that the adolescent's experiences of accessibility extended beyond the physical availability of YHS. While the physical presence of PHNs within the school environment and YHCs facilitated contact, accessibility also appeared to create a sense that sexual-related issues were legitimate topics for discussions within these settings. Thus, accessibility may not only enable help-seeking but also shape adolescents' perceptions of when, where, and for what reasons support can be sought. A previous study has similarly reported that accessibility influences adolescents' willingness to seek help, and that accessibility within the high-school setting is a key factor influencing adolescents' use of health services (42). School-based health services which are easy to access and free of charge, may help to reduce adolescents' concerns. This highlights the importance of the physical presence of PHNs as their availability facilitates timely support and reduces barriers to seeking help (42, 43). Accessibility is an essential factor in adolescents' use of healthcare (44). The findings also support the WHO's (14) emphasis on adolescents' right to accessible and acceptable health services and are consistent with international standards for youth-friendly service delivery (15). Given how society has developed and the many choices available in modern life, it is possible that increased uncertainty among adolescents may lead to a greater need for professional advice on how to achieve a fulfilling life (45). Therefore, considerations of privacy and discretion are important aspects of accessibility and may be just as crucial as practical factors, in reducing the threshold for seeking help. Accessible YHS appears to enhance adolescents' experience of manageability when they feel that they have sufficient resources and support from PHNs. This can help adolescents to cope better with the demands that result from the many impressions they encounter in their everyday lives. Taken together, accessibility is not only about the availability of services but also about ensuring that adolescents perceive support as relevant, worthwhile and available when needed.

The findings also highlight the importance of the relational qualities of encounters between adolescents and PHNs. Adolescents described conversations as informal and characterized by understanding, reassurance, support and a nonjudgmental attitude. Feeling listening to, taken seriously, and recognized as a whole person, appeared to facilitate communication and engagement. These findings underscore the importance of adopting a holistic approach that encompasses physical, mental, and spiritual wellbeing (46). Previous similar research suggests that adolescents value communication that reduces discomfort and tension and that professionals need to adapt their communication style to adolescents' needs in order to foster supportive rather than interrogative interaction (47, 48). In this, the findings resonate with Langaard's (45) description of adolescents' preference for helpers who demonstrate genuine attention, commitment and understanding. These relational qualities appear to be particularly important for adolescents' willingness to discuss sensitive issues and seek support. Previous research shows that respectful, caring and nonjudgmental interactions can reduce discomfort and facilitate trust in encounters with healthcare professionals (42, 47, 49). In contrast, experiences of being judged may undermine adolescents' confidence in health services and discourage engagement (50). Further, research suggests that trust can be strengthened when healthcare professionals attend to adolescents' emotional expressions, encourage collaborative dialogue, and work toward a shared understanding of their concerns (48). Together, these findings suggest that adolescents' engagement with YHS is shaped not only on the availability of support, but also on how the support is delivered and the quality of interaction with PHN.

Another finding of the study was that the adolescents expressed a desire for a more visible and active role of PHNs in sexual-related education and discussions. In particular, they value an increased presence of the PHNs in school settings to facilitate conversations about sexuality and strengthen the health literacy. The adolescents did not only view PHNs as healthcare providers to be contacted when problems arise, but also as trusted sources of knowledge who can contribute to learning and dialogue about sensitive topics. Adolescents appeared to value PHNs' professional expertise and their ability to provide reliable information in a non-judgmental manner, which may help reduce uncertainty and facilitate discussions. The importance of discussions with adolescents about sexuality has been highlighted, as young people are more likely to engage in risky sexual behavior than adults. A general change is underway in adolescents' sexuality, which can have significant consequences for health at both the individual and societal levels (11). This underscores the importance of PHNs taking a central role in discussing these topics. This can be particularly important in the school context, because schools play a significant role in promoting and protecting the health literacy and wellbeing of young people. Schools serve as important areas where young people acquire knowledge, develop socio-emotional skills such as self-regulation and resilience, and improve their critical thinking skills. These skills are considered fundamental to ensuring a healthy future (51). Sex education is essential for creating a safe environment where young people feel empowered to make informed choices about their bodies and relationships (52). The findings of the present study extend this knowledge by illustrating how adolescents themselves perceive PHNs as valuable contributors to such education and how guidance may be viewed as a trustworthy alternative to information obtained from the internet. In contrast, inadequate sex education, can lead to adolescents seeking information on the internet, which is often not the most reliable source (50). Through knowledge and education, adolescents can develop skills to manage their sexuality in a positive way. This includes protecting themselves against sexually transmitted infections and unwanted pregnancies, avoiding sexual situations they do not want, and seeking help from trusted people who can help when problems arise (3, 51). These findings are also consistent with Norwegian national guidelines (17), which emphasis that school health services should contribute to sexual health education in upper secondary schools. Strengthening the involvement of PHNs in classroom-based sexuality education may therefore represent an important strategy for promoting health literacy and supporting adolescents in navigating sexuality-related issues. Taken together, the presence of PHNs in educational settings may lower the threshold for future help-seeking. The PHNs role includes supporting adolescents in making informed decision about their health through dialogue guidance and health education which can actively strengthen and promote health decision making.

## 5 Implications for practice and need for further research

The findings indicate a growing need to enhance adolescents' health literacy concerning sexual and reproductive health. This can be supported by increased collaboration between SHS, teachers, and school administration. It is evident that PHNs become more actively incorporated into classroom-based sexual health education, with a view to improving accessibility, normalizing discussions on matters of a sexual nature and promoting learning in a safe environment. The results further suggest that adolescents value the presence of PHNs, indicating that discussions with relevant school authorities regarding the organization and increased visibility of SHS may be beneficial.

For future research, it would be beneficial to examine male adolescents' experiences of guidance and counseling on sexuality from PHNs, as well as developing targeted strategies for recruiting male adolescents to participate in studies. The study indicates that the gender of the PHN has an impact on female adolescents' experiences, and it is therefore important to explore whether this also affects male adolescents use of YHS.

## 6 Strengths and limitations

The methods discussion is based on the concepts of reliability, credibility, transferability, and authenticity (53), which are used to highlight the trustworthiness of a study. The study is considered to have maintained accuracy throughout the research process and provides clear explanations of how and why decisions were made. Overall, this contributes to strengthening the study's credibility (34).

PHNs participated in the recruitment by helping to identify adolescents with relevant experience, which strengthens the credibility of the study. The recruitment of male adolescents proved challenging. A significantly lower proportion of male adolescents than female adolescents use SHS and YHCs (17). Recruiting male adolescents in particular in clinical research is challenging, probably due to social roles and norms associated with gender (54). To recruit from hard-to-reach groups, it may be necessary to use innovative and time-consuming recruitment methods. Their perspective is often missing from research. Therefore, it is important to make a joint effort to develop projects that give underrepresented groups a voice (55).

Two adolescents aged 16–20 helped to develop the interview guide to ensure that it was appropriate and easy to understand. This user involvement is considered a strength (56), as it can increase the study's relevance and support practical application of the findings. A pilot interview with an adolescent was also conducted to test and refine the guide. Including the target group's perspectives strengthened user participation and improved the guide's relevance and quality.

When a sample has high information power, the number of interviews required is reduced (57). In this study, the eleven adolescents provided sufficient, relevant, and valuable data, which contributed to gaining a good basis for exploring characteristics and meaning in the data material. The opportunity to conduct digital interviews helped to include adolescents from different parts of Norway, which can strengthen the study's credibility by increasing the variation in content and diversity of the data material (34).

The interviews had an average duration of 18 min (range 12–30 min), which may be considered relatively brief given the potentially sensitive nature of the topic. However, the study aimed to explore experiences related to accessibility, guidance, and counseling, rather than participants' sexual health concerns themselves.

The inclusion of adolescents with different experiences in terms of age, gender, and residence in rural or urban areas, regardless of socioeconomic status, helps to illuminate the research question from multiple perspectives and strengthens the credibility of the study (53). However, male adolescents are not represented in the study due to recruitment challenges, which has resulted in skewed gender distribution. This may affect the transferability of the results. However, maximum diversity selection can help to ensure that adolescents with different backgrounds are represented in the sample (37). Although the study is based on 11 female adolescents and was conducted in a specific context, the insights may still be relevant and useful in other, similar contexts and can contribute to general knowledge.

Some experiences may have been overlooked, and other adolescents might have provided different perspectives. A limitation is the lack of control over what non-participants might have contributed. Self-selection bias may have occurred because participation was voluntary and the study may have attracted more motivated adolescents (33, 37). To mitigate this risk, recruitment was conducted through multiple channels, and variation in age, gender, ethnicity, and geographical location was sought (58).

A possible limitation of the study is the absence of member checks of the interview transcripts (59), which may reduce its credibility. However, the interviews were transcribed immediately afterwards, strengthening reliability because the transcriber was closely involved. This also made it easier to recall the interview context, including body language and expressions (37).

Three researchers were involved in and made decisions about the analysis, which reduced the risk of bias and strengthened the reliability of the study (37). To reduce the risk of losing meaning during the reduction and abstraction process, the most appropriate meaning units were chosen. Agreement was reached between all involved, which strengthens both the study's credibility and its authenticity (37, 53). At the same time, it is important to note that a text never has a single meaning; it is the researchers' interpretation of the most likely meaning that is presented (35).

There has been an awareness of the researcher's role, and active efforts have been made to minimize influence on participants' responses (33). Complete objectivity is currently challenging to achieve, as interpretations and biases can influence the results (60). To ensure credibility, strict methodological procedures were followed, including the development of an interview guide with open-ended questions, a neutral stance during data collection, careful handling of the data material, and a systematic analysis process (33–35, 37).

Reflexivity was maintained through continuous consideration of how the researchers' backgrounds and experiences might influence interpretations. Previous experiences as a PHN and working with adolescents and sexuality were acknowledged as part of the context, and prejudices were documented and actively considered in terms of how they might influence interpretations. Throughout the process, the researchers engaged in conscious reflection upon their own role, which helps to strengthen the quality and credibility of the study (33).

Rather than defining a specific population for the transfer of results, the inclusion criteria for this study are stated (37). It is therefore up to the reader to assess whether the results can be transferred to another context (35). A detailed description of the study's context, as well as a clear description of the data collection and analysis process, helps to strengthen transferability (53).

## 7 Conclusion

This study provides a comprehensive understanding of how adolescents' engagement with PHNs is shaped by the interaction between accessibility, the quality of relational aspects and the PHNs role in sexual health education. Together, these dimensions appear to contribute to the developments of adolescents' health literacy.

PHNs have been shown to play a pivotal role in the construction of a help-seeking climate thereby highlighting the significance of SHS and YHC, in the context of early health promotion and the development of adolescent's health literacy on sexuality. The findings indicate that YHS shape adolescents' perceptions of what kinds of issues are appropriate to bring forward.

The implication for practice involves an increased attention to how services are organized and delivered in everyday school contexts. This includes continuity of presence, low-threshold availability, and a focus on relational aspects that support trust-building. This implies that PHNs' role may benefit from being more visibly integrated, for more proactive engagement in the classrooms with adolescents rather than only consultations. Future research should focus on male adolescents' experiences of PHN support and how gender dynamics influence help-seeking and engagement with YHS.

## Data Availability

The data are not publicly available due to privacy or ethical restrictions. The data that support the findings of this study may be available on reasonable request from the corresponding author.
