# Supplementary material for: Adolescents' experiences of the public health nurse's accessibility, guidance, and counseling about sexuality: a qualitative study
Source: Front Public Health. 2026 Jul 23;14:1825174. doi: 10.3389/fpubh.2026.1825174 (PMC13442469; doi:10.3389/fpubh.2026.1825174)
Supplement: Supplementary file 1 [file Supplementary_File_1.pdf]

Interview guide

This study aimed to explore adolescents' experiences of the public health nurse´s accessibility, guidance, and counseling concerning sexuality.

Overall time frame approximately 30 min.

Start with:

Welcome, thank you for participating.

- Present interviews.
- Consent and information about the right to withdraw from the study without giving reasons.
- Written consent.

Information about the project:

- A little brief information about the background of the project.
- Through this interview we want to bring out your experiences, thoughts and impressions.
- This knowledge will help to develop the public health nursing service for adolescent.

Audio recording - ensuring confidentiality:

- We will use audio recordings during the interview, and the interview will be transcribed (written down). It will not be possible to trace back to specific individuals, and which statements are yours.
- The audio files will be kept secure and deleted after the study has been published.

Information about the interview situation:

- We will start by asking you some general questions about your background.
- We will then ask you some questions.
- If you need to talk after the interview, we can help you establish contact with a public health nurse in the municipality you belong to or give you contact information so that you can contact them directly.
- If there are any questions you do not want to talk about, that is of course fine.
- We are interested in your experiences, thoughts and impressions related to this topic.
- We ask that you do not share any personal health information and information about sexual relationships. This is to ensure your anonymity and privacy, as well as to comply with ethical guidelines in the study.
- To protect the privacy of the public health nurses, we would like to encourage you to speak in general about your experiences instead of mentioning specific people. In this way, we can ensure that no personal information is registered.

Now I will start the audio recorder and would like you to start by telling me your name, that you have read the information sheet, and whether you agree to participate in the research project.

Background questions:

Please tell me your:

- Age
- Gender
- Residence (rural/urban area)
- Current situation (education, job, other)

Main questions:

How did you realize that you could talk to a public health nurse about sexuality?

How did you experience the public health nurse's accessibility?

What significance does it have for you to have access to guidance and counseling about sexuality from a public health nurse?

Can you talk about receiving counseling and guidance about sexuality from a public health nurse?

How did you experience the public health nurse’s guidance and counseling?

What is important to you when you receive guidance and counseling?

How was the guidance and counseling with the public health nurse perceived?

What made it easy for you to talk to public health nurse?

What made it difficult for you to talk to public health nurse?

What did you find most helpful in the guidance and counseling you received?

How could the guidance and counseling about sexuality from public health nurses be different?

How do you think the gender of a public health nurse matter?

How do you think public health nurses can work around the topic of sexuality for adolescent?

Follow-up questions:

Can you tell me more ...

Can you elaborate ...

In what way ...

Closing:

Is there anything you would like to mention or add?

Thank you for joining us.
